# Health and education outcomes from adolescence to adulthood for young people with neurodisability and their peers: protocol for a population-based cohort study using linked hospital and education data from England

**DOI:** 10.1136/bmjopen-2025-100276

**Published:** 2025-03-18

**Authors:** Louise Macaulay, Jennifer Saxton, Tamsin Ford, Stuart Logan, Katie Harron, Ruth Gilbert, Ania Zylbersztejn

**Affiliations:** 1 Great Ormond Street Institute of Child Health, University College London, London, UK; 2 Department of Psychiatry, University of Cambridge, Cambridge, UK; 3 NIHR Applied Research Collaboration for the Southwest, University of Exeter Medical School, Exeter, UK; 4 NIHR Great Ormond Street Hospital Biomedical Research Centre, Great Ormond Street Hospital for Children NHS Foundation Trust, London, UK

**Keywords:** Adolescents, PUBLIC HEALTH, EPIDEMIOLOGY, Developmental neurology & neurodisability, Health Equity, STATISTICS & RESEARCH METHODS

## Abstract

**Abstract:**

**Introduction:**

Children and young people with neurodisability (conditions affecting the brain or nervous system, creating functional impairment, eg, autism, learning disabilities, epilepsy, cerebral palsy or attention-deficit/hyperactivity disorder) have more complex health and educational needs than their peers, contributing to higher healthcare use and special educational needs (SEN) provision. To guide policy and improve services, evidence is needed on how health and education support and outcomes change with age for adolescents with and without neurodisability.

**Methods and analysis:**

Using the Education and Child Health Insights from Linked Data (ECHILD) database, which links health and education data across England, we will follow adolescents from the start of secondary school (Year 7) into early adulthood. We will classify children with and without neurodisability recorded in hospital and education records before Year 7, compare their sociodemographic characteristics and describe trends in health and educational outcomes throughout secondary school. We will estimate rates of planned and unplanned healthcare contacts by year of age (11–22 years old), and we will examine changes in trends before, during and after transition to adult healthcare. We will also estimate the proportion of adolescents with school-recorded SEN provision and rates of school absences and exclusions by year of age (11–15 years old) for the two groups. We will explore variation in outcomes by neurodisability subgroup and sociodemographic characteristics and contextualise the findings using existing interview and survey data from children, young people and parents/carers generated in the Health Outcomes of young People throughout Education (HOPE) research programme.

**Ethics and dissemination:**

Ethics approval for analyses of the ECHILD database has been granted previously (20/EE/0180). Findings will be shared with academics, policymakers and stakeholders, and published in open-access journals. Code and metadata will be shared in the ECHILD GitHub repository.

**Strengths and limitations of this study**

- We use linked education and health data covering all of England, providing up to 12 years of follow-up and a large sample size for analyses stratified by neurodisability subgroups and sociodemographic factors.
- We use both education and health data to identify children and young people with neurodisability, to increase case ascertainment compared with using health data alone.
- We integrate lived experience evidence from children, young people and parents/carers from the Health Outcomes of young People throughout Education (HOPE) research programme, enhancing both the validity and depth of our findings and interpretation.
- The Education and Child Health Insights from Linked Data (ECHILD) database does not capture National Health Service healthcare outside acute hospital settings (such as primary care or additional support services provided in special schools), or education provided outside state-funded schools.
- Some children with neurodisability are likely to be omitted as not all are diagnosed or identified by services by the start of secondary school and others may be missed without linkage to other datasets, such as primary care or community paediatrics.

## Introduction

 Neurodisability refers to long-term congenital or acquired conditions that affect the brain and/or neuromuscular system and create functional limitations.^1^ It encompasses a broad range of conditions, which often coexist, including autism, learning disability, epilepsy, cerebral palsy, attention-deficit/hyperactivity disorder or genetic conditions (such as Down syndrome).^1^ Conditions that result in neurodisability are likely to affect a significant proportion of children and young people, but there is limited evidence on prevalence estimates for England.

Children and young people with neurodisability are among the most frequent and intensive users of health services and have disproportionately higher rates of healthcare utilisation including hospital admissions, accident and emergency (A&E) attendances and other healthcare contacts than their peers.^2^,^9^ Transition from paediatric to adult healthcare, usually between the ages of 16 and 18, is a particularly vulnerable period when support often becomes more fragmented, with longer waiting times and higher thresholds for accessing services.^10 11^ Changes in health management and provision of support during transition from paediatric to adult care may negatively impact young people’s health and result in unmet need and unplanned healthcare contacts.^12^,^14^ Understanding how healthcare support and unplanned healthcare contacts (such as emergency admissions or A&E visits) change with age can inform better co-ordination of healthcare services for adolescents.

Young people with neurodisability are also more likely to have additional learning needs and receive special educational needs (SEN) provision compared with their peers or young people with other complex chronic conditions.^4 5 15^ Although approximately 5% of children starting primary school from 2008/2009 to 2018/2019 had a neurodisability or associated high-risk condition recorded before age 5, they represent 10% of all children with any SEN provision and 30% of those with Education, Health and Care Plans^16^ (a more intensive provision arranged and partly funded by local authorities^17^). Educational trajectories for young people with neurodisability are often interrupted due to school absences and/or exclusions,^45 15 18^,^22^ which likely contributes to lower school attainment^4 5 23^ and fewer young people with neurodisability completing secondary education.^5 24 25^ Education is a key social determinant of health and can impact young people’s future health and independence as adults.^26 27^ Better understanding of trends in school participation and SEN provision by age for young people with neurodisability can support better co-ordination between health and education services and may inform policies to increase school participation.

Despite high usage of special educational support and specialist healthcare by children and young people with neurodisability, there is a lack of evidence on changes in planned support (eg, planned hospital care, SEN provision) and adverse outcomes over time (eg, unplanned hospital admissions, school absences and exclusions). Further, there is limited evidence on how the needs of young people with neurodisability change with age. This study is the first step to understanding these inter-relationships. Using the Education and Child Health Insights from Linked Data (ECHILD) database,^28^ we will conduct longitudinal analyses of linked healthcare and education administrative data to describe how planned support and adverse outcomes change with age from adolescence to young adulthood. We hypothesise that as levels of proactive support decline, some adverse outcomes may increase. These descriptive analyses will inform future study designs evaluating the impact of support on long-term outcomes.

This Programme Development Grant (PDG) builds on previous work from the Health Outcomes of young People throughout Education (HOPE) research programme (referred to as the HOPE Study throughout), which aimed to examine variation in SEN provision and its impact on health and education outcomes for children with specific health conditions,^16^ including neurodisability.^29 30^ In this HOPE PDG, we extend the follow-up period from primary school into young adulthood and integrate new quantitative evidence on adolescent support and outcomes with findings from HOPE’s interviews and surveys from young people and parents/carers. This HOPE PDG began in August 2024 and is expected to end in January 2026. The research programme is ongoing and groundwork for developing the study cohort, including data cleaning, validation and the creation of coding algorithms to define the cohort for all objectives has begun.

### Aim and objectives

The overall aim of this study is to describe how planned support (planned hospital care, SEN provision) and adverse outcomes (unplanned hospital care, absences or exclusion from school) change from adolescence to adulthood among young people with neurodisability and their peers. Our research seeks to answer the question: How do health and education outcomes from adolescence to adulthood vary among young people with neurodisability and their unaffected peers?

The specific objectives are to:

1. Characterise cohorts of adolescents with neurodisability at secondary school entry using whole-country linked health and education records in England.
2. Describe trends in planned support and adverse outcomes for adolescents and young adults with and without neurodisability in:
  - The healthcare setting (focusing on planned and unplanned hospital contacts).
  - Secondary schools (focusing on SEN provision, school attendance and exclusions).

## Methods and analysis

### Study design

We will develop a cohort of secondary school aged pupils using the ECHILD database, with longitudinal follow-up across health and education. Although all proposed analyses are descriptive, we will design the studies using the target trial emulation (TTE) framework,^31 32^ in line with the approach taken in the HOPE Study.^16^ The TTE framework, typically used to guide analyses of observational data to examine causal effects, involves two key steps: (1) specifying an ideal pragmatic randomised controlled trial (RCT) to address the research question of interest, and (2) defining how this RCT can be emulated as closely as possible using observational data. In the HOPE PDG, we adapted the TTE framework to guide the design of descriptive studies. This involved specifying the target population-based study, describing how it can be emulated using ECHILD data and identifying potential sources of bias (see online supplemental appendix for details of TTE for this programme).^32^ This approach helps assess potential biases arising from the use of observational data and identifies ways to mitigate them at the design stage.

### Data sources and linkage

The ECHILD database links routinely collected administrative data on health and education in England.^28^ Information on health comes from Hospital Episode Statistics (HES). HES is a national database that includes dated information on all National Health Service (NHS) acute hospital care (including inpatient, outpatient and A&E records) and mortality data.^33^ Education records are collated in the National Pupil Database (NPD) and include information from all state-funded schools on children and young people’s registrations in schools, education attainment scores, absences, exclusions and SEN provision.^34^ As part of the HOPE Study, we also enhanced the ECHILD database using open-source data on school characteristics (eg, type of school).^35^

### Study population and follow-up

We will develop a cohort of secondary school aged adolescents enrolled in state-funded schools in Year 7 (usually aged 11–12 years old) between 2008/2009 and 2014/2015. We will exclude young people who are 2 or more years outside of their expected age for their school year.

**Figure 1:**
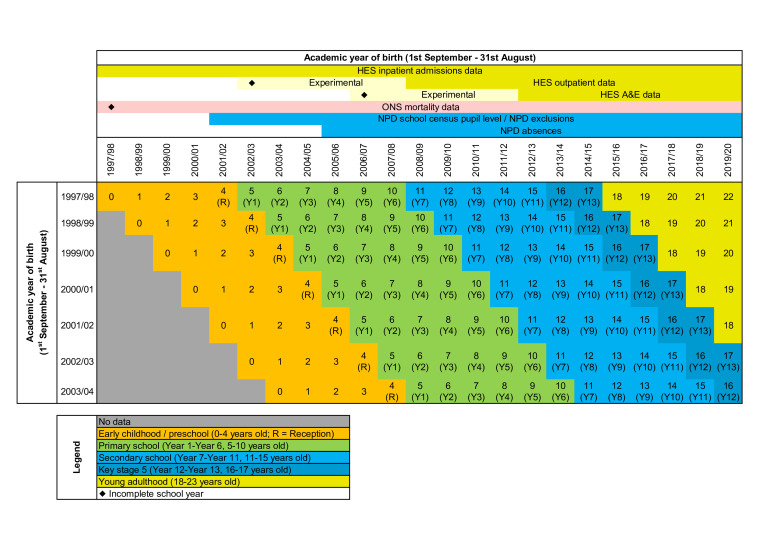
Overview of the study cohorts, their expected age of entry into each school year and the available data. A&E, accident and emergency; HES, Hospital Episode Statistics; NPD, National Pupil Database; ONS, Office for National Statistics.

Adolescents will be followed up from 1 February (after the spring school census) in Year 7 through to Year 11 (the end of secondary school, usually aged 15–16 years old) in education records, and until aged 22 in healthcare records (due to data availability) (figure 1). Education outcomes will be measured until the end of term 1 of the 2019/2020 academic year (31 December 2019) in education records, the last full academic term before the COVID-19 pandemic. Healthcare outcomes will be measured up until 1 March 2020, just before the first COVID-19 lockdown in England, as the pandemic impacted access to school and the frequency of hospital contacts captured in ECHILD data.^36 37^

### Defining key study measures

#### Defining neurodisability in administrative records

We propose two complementary approaches based on health and education data recorded in HES and in NPD to identify adolescents with neurodisability before the start of secondary school (Year 7), ensuring that all exposures are measured before the outcomes. Ensuring exposures are measured before outcomes reduces the risk of reverse causality, where the outcome might influence the exposures. Although this is descriptive work, maintaining this temporal sequence helps establish a clear direction of influence. Throughout the protocol, we refer to children with neurodisability recorded in HES and NPD as ‘children with neurodisability’, although we note that some children with neurodisability are likely to be omitted in the absence of linkage to other datasets (including primary care or community paediatrics) or because their difficulties have not been identified by any service.

We will use a phenotyping algorithm for indicating conditions with high risk of neurodisability from diagnostic data recorded in HES admitted patient care records developed as part of the HOPE Study.^16^ This list includes conditions such as cerebral palsy, epilepsy, autism, learning disabilities, muscular dystrophies and other neurologic conditions associated with high risk of functional impairment (such as hydrocephalus, brain tumours or indicators of infection or inflammation of the brain).^1 8 12 38 39^ We will use diagnostic data from all hospital admissions before entry to Year 7. We will examine recording of included conditions in adolescents and consult expert clinicians to derive clinically meaningful subgroups of conditions with comparable health and educational needs and functional impairment. These subgroups could include: (1) intellectual and developmental disorders; (2) autism spectrum disorder; (3) cerebral palsy; (4) epilepsy; and (5) genetic and chromosomal conditions.

As adolescents with neurodisability receive much of their care outside of secondary healthcare settings (ie, in community paediatrics, primary care), some conditions are likely to be under-reported in hospital records (eg, autism or hyperkinetic disorders).^12 40^ We will therefore complement information from hospital records with information on types of need for SEN provision recorded during primary school to increase case ascertainment. This approach has been used previously in linked health and education data from Scotland^18 41^ and in studies based on education data only in England.^20 42 43^ We will examine recording of likely autism and learning disabilities using information on primary and secondary type of need for SEN provision in primary school records (Years 1–6, aged 5/6–10/11 years old). Learning disabilities will be indicated by combining three types of SEN (categorised in an ascending hierarchy): (1) profound and multiple learning difficulties (where children are likely to have severe and complex learning difficulties as well as a physical disability or sensory impairment); (2) severe learning difficulties (where children are likely to need support in all areas of the curriculum and associated difficulties with mobility and communication); and (3) moderate learning difficulties.^17^ We only use information on type of need collected prior to 2014/2015 education reforms (see figure 1),^17^ ensuring consistency of coding of SEN need over time.

#### Outcome: mortality

As part of descriptive work towards objective 1, we will examine all-cause mortality rates in young people with neurodisability compared with their peers. Mortality will be compared based on the data source used to identify neurodisability (HES vs NPD) and for children with different types of neurodisability. Information on deaths will be obtained from linked Office for National Statistics (ONS) mortality records and supplemented with information on in-hospital deaths recorded in HES. Children will be followed from 1 February of Year 7 until death or 1 March 2020, whichever occurred first.

#### Outcome: planned and unplanned healthcare contacts

We will focus on planned and unplanned healthcare contacts which can be measured in HES. The primary outcomes of interest are planned and unplanned hospital admission rates, defined as the number of admissions per 100 person-years at risk per year of age. We will look at admissions overall and by reason for admission (using recorded primary diagnoses). We will compare the most common three-character International Classification of Diseases version 10 (ICD-10) codes for primary diagnoses recorded for adolescents with neurodisability versus their peers by each year of age to determine which reasons for planned/unplanned admissions should be considered for further analyses.

Person-years at risk will be calculated as time from start of Year 7 until the end of follow-up (23^rd^ birthday, death or 1 March 2020, whichever occurred first). We will exclude time spent in hospital during hospital admissions from person-years at risk and split follow-up by year of age to enable derivation of age-specific rates. Secondary outcomes will include A&E department contact rates (defined as the number of days with at least one A&E contact divided by person-years at risk) and outpatient department (OPD) attendances (defined as the number of days with at least one OPD attendance divided by person-years at risk). While classified as ‘unplanned’, A&E attendances are not necessarily adverse events, as they may represent appropriate healthcare access and timely intervention. Due to the uncertain quality of A&E data prior to 1 April 2012 (as data were deemed ‘experimental’, see figure 1), we will set the follow-up start for this outcome to 1 April 2012.

#### Outcome: educational outcomes

We will describe changes in educational support (measured as school-recorded SEN provision) and school participation (measured by absences and exclusions from school) per year of age across secondary school (between start of Year 7 and end of Year 11, or until loss of follow-up, death or 31 December 2019, whichever occurred first) for adolescents with neurodisability and unaffected peers. Our primary focus will be on persistent absences. Secondary outcomes will be medical/non-medical absences and exclusions. All outcomes are defined in table 1.

**Table 1:** Definitions of educational outcomes

Outcomes		Definition	Dataset(s)	Measured
**School participation**	Persistent absentees	Pupils who miss 10% or more of their possible sessions^45^	NPD School Census	Year 7–Year 11 (11/12–15/16 years old)
	Absence for medical reasons	Authorised/unauthorised absences for doctor/dentist appointments or illness*		
	Absences for non-medical reasons	Authorised/unauthorised absences for all other reasons than medical*		
	Exclusions	Pupils with at least one suspension (fixed-period exclusion) or permanent exclusion in the given school year		
**Educational support**	SEN provision (by type)	SEN provision will be grouped in the following descending hierarchy: No SEN provision SEN support in mainstream school EHCP in mainstream school Enrolment in specialist provision		

* We will use the total number of possible sessions rather than pupils as denominator.

EHCP, Education, Health and Care PlanNPD, National Pupil Database; SEN, special educational needs

NPD data include some information about post-16 educational outcomes (such as special school attendance until 19 years old or education, employment or training at 17–18 years from National Client Caseload Information System),^34^ and we will explore the feasibility of using these data to examine educational trajectories of adolescents with neurodisability after secondary school.

### Additional covariates of interest

We will derive a set of sociodemographic characteristics recorded in or before inception to the cohort in Year 7 (table 2). These include child characteristics (such as gender, ethnic group) and socioeconomic factors (such as area-level deprivation or eligibility for free school meals) which will be obtained from NPD school census. We will use information about hospital admissions before the start of Year 7 to derive indicators of complexity of health needs: coexisting chronic conditions (using all data prior to Year 7) and frequency of healthcare contacts in Year 6 (table 2).

**Table 2:** Sociodemographic characteristics at baseline for pupils in the secondary school cohort

Variables		Definition	Dataset(s)	Measured
**Child characteristics**	Year of birth	Grouped as academic years from 1 September to 31 August	NPD School Census	Year 7 (aged 11/12 years old)
	Month of birth	January to December		
	Gender	Young person’s parent/carer or self-reported gender		
	First language	Young person’s first language (English or other)		
	Ethnic group	Young person’s parent/carer or self-reported ethnicity		
**Socioeconomic factors**	Index of Multiple Deprivation quintile	Quintile of area-level deprivation based on young person’s residential address		
	Free school meals	Eligibility for free school meals (yes or no)		
**Indicators of complexity of health needs**	Chronic conditions (excluding neurodisability)	Presence of chronic conditions^46^ indicated from diagnoses recorded during any hospital admission before the start of secondary school	HES APC	Aged 0–11 years old
	Frequency of healthcare contacts	Number of planned/unplanned hospital admissions, accident and emergency (A&E) attendances and outpatient appointments	HES APC,OP,A&E	Aged 10–11 years old (Year 6)

APC, admitted patient care; HES, Hospital Episode Statistics; NPD, National Pupil Database; OP, outpatient care

### Statistical analysis

#### Objective 1: descriptive analysis

We will derive point prevalence of neurodisability at the start of Year 7 (defined as the number of pupils with a hospital or school record of neurodisability at any point before they entered Year 7 divided by the number of pupils enrolled in Year 7), overall and by neurodisability subgroup. Children with neurodisability are more likely to live in more socioeconomically disadvantaged households, experiencing multiple social determinants of health which may affect their underlying health needs as well as their access to support services. We will describe numbers and percentages for adolescents according to baseline sociodemographic characteristics (table 2) to understand potential overlaps in factors contributing to health inequalities. NPD and HES are likely to capture adolescents with different levels of health need, we will therefore also describe the overlap and baseline characteristics of adolescents with neurodisability captured in education and health datasets. We will also compare mortality rates in adolescents with neurodisability, overall and according to data source used as a crude measure of complexity of health needs.

ECHILD does not capture termly data on children and young people educated in the private sector (approximately 7% of pupils in England each year).^34^ We will estimate the number of adolescents lost at the start of Year 7 due to being educated outside of state-funded schools. This is defined as the difference in the number of pupils ever enrolled in state-funded primary school and alive in Year 7 and the number of adolescents enrolled in Year 7.

#### Objective 2a: trends in planned and unplanned admissions

We will estimate rates of planned and unplanned healthcare contacts by age for adolescents with neurodisability (overall and by subgroup) and their peers. We will use Poisson or negative binomial regression models to estimate rate ratios for healthcare contacts for adolescents with neurodisability versus peers. We will assess whether trends in provision of planned support and unplanned healthcare contacts change before, during and after transition to adult healthcare (at ages 11–15, 16–18 and 19–22 years old, respectively) by including three binary indicators (intercepts) and three linear splines (slopes) for each age category (unless different specification is more appropriate on visual examination of figures). The intercepts indicate healthcare contact rates at baseline (11, 16 and 19 years old), while the slopes describe the rate of change as age increases. Models will include academic year of birth (to account for possible cohort effects) and we will also account for repeated measurements within pupils over time (eg, by allowing for a random effect by individual). We will test whether trajectories of healthcare use differ between adolescents with neurodisability and their peers by adding interaction terms between neurodisability and age variables.

We will also examine differences in support and outcomes among adolescents with neurodisability according to sociodemographic characteristics and school placement type (mainstream/special) at the start of Year 7. We will calculate and plot rates of planned and unplanned healthcare contacts by pupil characteristics, socioeconomic factors and school type for adolescents with and without neurodisability.

#### Objective 2b: trends in educational support, school attendance and exclusions

We will calculate and plot the proportion of pupils experiencing each educational outcome (defined as the number of pupils with at least one recording of the outcome divided by the number of pupils on school roll at the start of the given school year) by year of age. We will use a Poisson or logistic regression model to quantify the differences between adolescents with neurodisability and their peers. The models will include age, birth year and an indicator of neurodisability, and we will account for repeated measures per child. We will also graphically quantify changes in proportions of adolescents experiencing each outcome according to neurodisability subgroup and sociodemographic characteristics (separately for adolescents with neurodisability and their peers).

Analyses carried out for objectives 2a and 2b will reveal whether support and adverse outcomes across health and education follow similar trajectories with age for adolescents with neurodisability and their peers and illustrate potential inequalities in support and adverse outcomes to inform design of further studies.

### Patient and public involvement

This research programme builds on past involvement of children, parents/carers and organisations as part of the HOPE Study and the ECHILD project. Our research objectives were informed by themes identified in involvement to date.^44^ Parents/carers and young people felt that communication between health and education settings is often insufficient, with teachers often unaware of young people’s health needs. Young people who missed school because of health needs voiced the need for more adjustments to support return to school, for example, tailored timetables and personalised sessions. Young people also voiced that school changes and taking examinations are a source of stress, and additional support around these critical periods was needed. A preference was expressed for a broader curriculum, more flexible teaching styles and a move back towards coursework counting towards assessment. We also heard from patient groups about poor recognition of mental health needs, particularly in secondary school, among children with learning disability. Finally, parents/carers and young people supported further research around transition to adult services, voicing that co-ordination of care often fails during transition from paediatric to adult care, impacting on young people’s health and well-being. These insights informed the study design, particularly its focus on examining both healthcare contacts and school participation, including changes in attendance patterns and healthcare use during the transition to adult services for young people with neurodisability.

Parents/carers and young people will continue to be involved throughout the research programme. We plan at least three meetings with young people and parents/carers to gain feedback and insights on the design of our study protocol and discuss findings and implications for policy and practice. In brief, we will first present our planned analyses and discuss research priorities (eg, which educational outcomes are of most importance to families, what can we measure, are there cause-specific hospital admissions that we should focus on further?). Second, we will return to the same groups to share our findings and discuss how different pathways through health and education seen in the data relate to their experiences and what support might be important.

We will also organise a series of seminars with researchers from the HOPE Study to facilitate knowledge exchange. The HOPE Study used mixed methods (eg, interviews, surveys and document analysis) to understand variation in the underlying processes of identification, assessment and provision of SEN support across geographic areas in England and how these processes are experienced by families. Our findings will provide an overview of how SEN provision varies with age at a national level. Findings from HOPE Study on factors associated with variation in SEN provision will help interpret our findings on SEN provision, as ECHILD contains records indicating provision for SEN, but no information about whether any provision was received, when or its type or quality.

### Ethics and dissemination

#### Ethics

Permissions to use linked, de-identified data from HES and the NPD were granted by the Department for Education (DfE) (DR200604.02B) and NHS Digital (DARS-NIC-381972). Ethical approval for the ECHILD project was granted by the National Research Ethics Service (17/LO/1494), NHS Health Research Authority Research Ethics Committee (20/EE/0180 and 21/SW/0159) and UCL Great Ormond Street Institute of Child Health’s Joint Research and Development Office (20PE06).

#### Dissemination

ECHILD data are not shared publicly in line with data-sharing agreements with NHS Digital and DfE. ECHILD can be accessed by accredited researchers through application via the ECHILD team (www.echild.ac.uk) and the Research Accreditation Panel. We will publish our methods and code to enable others to reproduce and extend our analyses using ECHILD. Metadata and code relating to this study will be signposted on the study website and made available in the ONS secure environment and in a code repository (including the ECHILD GitHub page: https://github.com/UCL-ECHILD).

Dissemination will include the presentation of preliminary findings to academics, HOPE and policy stakeholders (including analysts at ONS, DHSC and DfE). Seminars and consultations with parents/carers and young people involved in patient and public involvement activities for this project will also be held throughout the study. Final outputs will include peer-reviewed journal articles, the final study report to funder and infographics for non-academic audiences. The cohort established for this study will set the groundwork for further studies of long-term health and education outcomes for adolescents with neurodisability and chronic conditions, such as educational and post-16 attainment.

## supplementary material

10.1136/bmjopen-2025-100276 online supplemental table 1

## References

[1] Morris C, Janssens A, Tomlinson R (2013). Towards a definition of neurodisability: a Delphi survey. Dev Med Child Neurol.

[2] Arim RG, Miller AR, Guèvremont A (2017). Children with neurodevelopmental disorders and disabilities: a population-based study of healthcare service utilization using administrative data. Dev Med Child Neurol.

[3] Carter B, Bennett CV, Jones H (2021). Healthcare use by children and young adults with cerebral palsy. Dev Med Child Neurol.

[4] Fleming M, Fitton CA, Steiner MFC (2017). Educational and Health Outcomes of Children Treated for Attention-Deficit/Hyperactivity Disorder. JAMA Pediatr.

[5] Fleming M, Fitton CA, Steiner MFC (2019). Educational and health outcomes of children and adolescents receiving antiepileptic medication: Scotland-wide record linkage study of 766 244 schoolchildren. BMC Public Health.

[6] Yuan J-X, McGowan M, Hadjikoumi I (2017). Do children with neurological disabilities use more inpatient resources: an observational study. Emerg Themes Epidemiol.

[7] Cohen E, Berry JG, Camacho X (2012). Patterns and costs of health care use of children with medical complexity. Pediatrics.

[8] Bayer ND, Hall M, Li Y (2022). Trends in Health Care Use and Spending for Young Children With Neurologic Impairment. Pediatrics.

[9] The National Confidential Enquiry into Patient Outcome and Death (2018). Each and Every Need London.

[10] Brown M, Macarthur J, Higgins A (2019). Transitions from child to adult health care for young people with intellectual disabilities: A systematic review. J Adv Nurs.

[11] Shanahan P, Ollis L, Balla K (2021). Experiences of transition from children’s to adult’s healthcare services for young people with a neurodevelopmental condition. Health Soc Care Community.

[12] Zylbersztejn A, Stilwell PA, Zhu H (2023). Trends in hospital admissions during transition from paediatric to adult services for young people with learning disabilities or autism: Population-based cohort study. Lancet Reg Health Eur.

[13] Wijlaars LPMM, Hardelid P, Guttmann A (2018). Emergency admissions and long-term conditions during transition from paediatric to adult care: a cross-sectional study using Hospital Episode Statistics data. BMJ Open.

[14] Jarvis S, Flemming K, Richardson G (2022). Adult healthcare is associated with more emergency healthcare for young people with life-limiting conditions. Pediatr Res.

[15] John A, Friedmann Y, DelPozo-Banos M (2022). Association of school absence and exclusion with recorded neurodevelopmental disorders, mental disorders, or self-harm: a nationwide, retrospective, electronic cohort study of children and young people in Wales, UK. Lancet Psychiatry.

[16] Zylbersztejn A, Lewis K, Nguyen V (2023). Evaluation of variation in special educational needs provision and its impact on health and education using administrative records for England: umbrella protocol for a mixed-methods research programme. BMJ Open.

[17] (2015). Department for Education. Special educational needs and disability code of practice: 0 to 25 years.

[18] Fleming M, Salim EE, Mackay DF (2020). Neurodevelopmental multimorbidity and educational outcomes of Scottish schoolchildren: A population-based record linkage cohort study. PLoS Med.

[19] Fleming M, Fitton CA, Steiner MFC (2020). Educational and health outcomes of children and adolescents receiving antidepressant medication: Scotland-wide retrospective record linkage cohort study of 766 237 schoolchildren. Int J Epidemiol.

[20] Hatton C (2018). School absences and exclusions experienced by children with learning disabilities and autistic children in 2016/17 in England. *TLDR*.

[21] Tanya Lereya S, Cattan S, Yoon Y (2023). How does the association between special education need and absence vary overtime and across special education need types?. Eur J Spec Needs Educ.

[22] Black LI, Zablotsky B (2018). Chronic School Absenteeism Among Children With Selected Developmental Disabilities: National Health Interview Survey, 2014-2016. Natl Health Stat Report.

[23] Lystad RP, McMaugh A, Herkes G (2022). The impact of childhood epilepsy on academic performance: A population-based matched cohort study. Seizure: European Journal of Epilepsy.

[24] Sentenac M, Lach LM, Gariepy G (2019). Education disparities in young people with and without neurodisabilities. Dev Med Child Neurol.

[25] Rasalingam A, Brekke I, Dahl E (2021). Impact of growing up with somatic long-term health challenges on school completion, NEET status and disability pension: a population-based longitudinal study. BMC Public Health.

[26] Viner RM, Ozer EM, Denny S (2012). Adolescence and the social determinants of health. The Lancet.

[27] Raghupathi V, Raghupathi W (2020). The influence of education on health: an empirical assessment of OECD countries for the period 1995-2015. Arch Public Health.

[28] Mc Grath-Lone L, Libuy N, Harron K (2022). Data Resource Profile: The Education and Child Health Insights from Linked Data (ECHILD) Database. Int J Epidemiol.

[29] Cant A, Zylbersztejn A, Gimeno L (2024). Primary school attainment outcomes in children with neurodisability: Protocol for a population-based cohort study using linked education and hospital data from England. *NIHR Open Res*.

[30] Gimeno L, Zylbersztejn A, Cant A (2024). Planned and unplanned hospital admissions and health-related school absence rates in children with neurodisability: Protocol for a population-based study using linked education and hospital data from England. *NIHR Open Res*.

[31] Hernán MA, Robins JM (2016). Using Big Data to Emulate a Target Trial When a Randomized Trial Is Not Available. Am J Epidemiol.

[32] Hernán MA, Wang W, Leaf DE (2022). Target Trial Emulation: A Framework for Causal Inference From Observational Data. JAMA.

[33] Herbert A, Wijlaars L, Zylbersztejn A (2017). Data Resource Profile: Hospital Episode Statistics Admitted Patient Care (HES APC). Int J Epidemiol.

[34] Jay MA, McGrath-Lone L, Gilbert R (2019). Data Resource: the National Pupil Database (NPD). Int J Popul Data Sci.

[35] Department for Education (2024). Get Information about Schools (GIAS).

[36] Etoori D, Harron KL, Mc Grath-Lone L (2022). Reductions in hospital care among clinically vulnerable children aged 0-4 years during the COVID-19 pandemic. Arch Dis Child.

[37] Mc Grath-Lone L, Etoori D, Gilbert R (2022). Changes in adolescents’ planned hospital care during the COVID-19 pandemic: analysis of linked administrative data. Arch Dis Child.

[38] Jarvis SW, Livingston J, Childs A-M (2018). The impact of neurological disorders on hospital admissions for children and young people: a routine health data study. *Int J Popul Data Sci*.

[39] Berry JG, Poduri A, Bonkowsky JL (2012). Trends in resource utilization by children with neurological impairment in the United States inpatient health care system: a repeat cross-sectional study. PLoS Med.

[40] Zylbersztejn A, Cant A, Gimeno L (2024). Phenotyping neurodisability in hospital admissions records in England: a descriptive study of a national birth cohort. Prep.

[41] Smith GS, Fleming M, Kinnear D (2020). Rates and causes of mortality among children and young people with and without intellectual disabilities in Scotland: a record linkage cohort study of 796 190 school children. BMJ Open.

[42] Emerson E (2012). Deprivation, ethnicity and the prevalence of intellectual and developmental disabilities. J Epidemiol Community Health.

[43] Roman-Urrestarazu A, van Kessel R, Allison C (2021). Association of Race/Ethnicity and Social Disadvantage With Autism Prevalence in 7 Million School Children in England. JAMA Pediatr.

[44] ECHILD (2024). Engaging with the public. echild educ. child health insights linked data. https://www.echild.ac.uk/engagements.

[45] Department for Education (2024). Pupil absence in schools in england, autumn and spring term 2023/24. https://explore-education-statistics.service.gov.uk/find-statistics/pupil-absence-in-schools-in-england.

[46] Hardelid P, Dattani N, Gilbert R (2014). Estimating the prevalence of chronic conditions in children who die in England, Scotland and Wales: a data linkage cohort study. BMJ Open.

