# Supplementary material for: Health and education outcomes from adolescence to adulthood for young people with neurodisability and their peers: protocol for a population-based cohort study using linked hospital and education data from England
Source: BMJ Open. 2025 Mar 18;15(3):e100276. doi: 10.1136/bmjopen-2025-100276 (PMC11927417; doi:10.1136/bmjopen-2025-100276)
Supplement: online supplemental table 1 [file bmjopen-15-3-s001.docx]

Online supplementary appendix: Summary protocol: Emulating a population-based study to describe health and education outcomes in young people with neurodisability using ECHILD data

| Protocol component | Target population- based study specification (i.e. the “ideal” study) | Emulation study (i.e. this study) | Potential biases | Mitigation strategies |
| --- | --- | --- | --- | --- |
| Study design | Prospective observational cohort study | Retrospective analyses of prospectively collected linked administrative health and education records | Data is collected for administrative purposes therefore some useful/ important data items may be missing; there may be potential data entry errors.  Hospital admission data are collected for financial reimbursement purposes; the depth of clinical coding increases over time. | Where possible we will derive additional covariates from available data (e.g. chronic conditions) and combine information from HES and NPD to minimise missing data/data entry errors.  We will adjust all analyses for year of birth. We will examine mean and median number of recorded diagnoses per record per calendar year. We will examine prevalence of conditions by birth years. |
| Eligibility criteria | Enrolled in school in Year 7 between 2008/09 and 2014/15 in England | Have a record in NPD indicating enrolment in a state-funded school in England in Year 7 (or aged 11 if not following the national curriculum) between 2008/09 and 2014/15. Linked to a HES record | Selection bias due to linkage errors, and lack of data on pupils educated outside of state-funded schools. | We will compare characteristics of pupils with and without linked HES data.  We will estimate the number of adolescents lost at the start of Year 7 due to being educated outside of state-funded schools. |
| Exposure | Clinical diagnosis of neurodisability before the start of secondary school (year 7) | Neurodisability or associated high-risk condition (based on phenotyping algorithms from HOPE) recorded as a diagnosis in HES during any hospital admission before 1^st^ September of Year 7  Learning disability or autism indicated in NPD (as any type of SEN need) before Year 7 | Underreporting of neurodisability due to some children not being diagnosed or identified by services by Year 7, and  in hospital admission records in absence of linkage to other datasets (including primary care or community paediatrics).  Misclassification of neurodisability (not all children with considered conditions will have functional impairment) | We will indicate children with conditions associated with neurodisability in ≥50% of cases (based on clinical input) to increase case ascertainment and additionally use school records to capture children who are flagged by schools to have a likely neurodisability.  We will carry out additional validation of neurodisability phenotype: we will describe sociodemographic characteristics of children and young people with and without neurodisability, the prevalence of different neurodisability conditions by age at first record in HES and by sociodemographic characteristics, clustering of conditions and the key outcomes for most common conditions for comparison with published figures and clinical expectation. |
| Analyses of health outcomes | | | | |
| Outcome | Planned and unplanned healthcare contacts | Planned secondary healthcare contacts:  planned hospital admission rates and outpatient department (OPD) attendance rates  Unplanned secondary healthcare contacts:  unplanned hospital admission rates and A&E department contact rates | We are limited to secondary healthcare and do not have linked primary care, community paediatrics or mental health services data to understand trends in overall healthcare utilisation | We will examine rates of health-related school absences as additional measure of healthcare contacts not captured in HES.  We will seek findings from literature on trends in primary care contacts. |
| Follow-up period | Follow-up from secondary school entry (1^st^ September of Year 7) until earliest of 1^st^ March 2020, death, or loss of follow-up. | Follow-up from 1^st^ September of Year 7 until death or 1^st^ March 2020 (whichever occurred first).  We will exclude time spent in hospital during hospital admissions from person-years at risk | We are not able to account for migration out of England as these data are not available in ECHILD. |  |
| Target of estimation | Difference in healthcare contact rates over time between children with and without neurodisability, accounting for sociodemographic factors | Difference in secondary healthcare contact rates over time between children with and without hospital- or school-recorded neurodisability, accounting for sociodemographic factors |  |  |
| Analysis plan | Poisson or negative binomial regression models to estimate healthcare contact rate ratios per year of age; models will adjust for sociodemographic factors and account for repeated measures over time | Poisson or negative binomial regression models to estimate healthcare contact rate ratios per year of age; models will adjust for sociodemographic factors and account for repeated measures over time |  |  |
| Analysis of education outcomes | | | | |
| Outcomes | SEN provision reported by teaching staff and local authorities, absences and exclusions reported by teaching staff | School-recorded SEN provision,  termly number of missed school sessions (2 per day) out of all sessions available and termly exclusions | School-recorded SEN provision does not provide indication if any provision as actually received, or what it involved.  It is not required for schools to record reason for absence. There may be school-level variation and changes over time in how reasons for absence are recorded. | We will clearly outline the limitations of our measure of “SEN provision”  Findings will be contextualised using interview and survey findings from HOPE research programme.  We will report overall absence rate. For analyses involving reasons for absence, we will visually assess trends in recording by calendar year to validate the data prior to analyses. |
| Follow-up period | Follow-up from start of secondary school (1^st^ September of Year 7) until the earliest of end of secondary school (Year 11), death, or loss of follow-up | Follow-up from 1^st^ September in Year 7 until the end of Year 11 (the end of secondary school, usually aged 15 to 16 years old), 31^st^ December 2019 (end of term 1 of 2019/20), death or moving out of state-funded school (no record in NPD census) | We are not able to account for migration out of England as these data are not available in ECHILD. |  |
| Target of estimation | Risk/odds ratio for having SEN provision/being persistently absent for children with neurodisability vs peers; accounting for additional health needs and sociodemographic factors | Risk/odds ratio for having SEN provision/being persistently absent for children with neurodisability vs peers; accounting for additional health needs and sociodemographic factors |  |  |
| Analysis plan | Poisson or logistic regression models to estimate healthcare contact rate ratios per year of age; models will adjust for health needs and sociodemographic factors and account for repeated measures over time | Poisson or logistic regression models to estimate healthcare contact rate ratios per year of age; models will adjust for health needs and sociodemographic factors and account for repeated measures over time |  |  |

ECHILD = Education and Child Health Insights from Linked Data; HES: Hospital Episode Statistics; HOPE = Health Outcomes of young People throughout Education; NPD: national pupil database, SEN = special educational needs
